# Systematic analysis of negative and positive feedback loops for robustness and temperature compensation in circadian rhythms

**DOI:** 10.1038/s41540-023-00268-7

**Published:** 2023-02-11

**Authors:** Suchana Chakravarty, Christian I. Hong, Attila Csikász-Nagy

**Affiliations:** 1 Faculty of Information Technology and Bionics, Pázmány Péter Catholic University, Budapest, Hungary; 2 Department of Pharmacology & Systems Physiology, University of Cincinnati, Cincinnati, OH USA; 3 Division of Developmental Biology, Cincinnati Children’s Hospital Medical Center, Cincinnati, OH USA

**Keywords:** Oscillators, Biochemical networks

## Abstract

Temperature compensation and robustness to biological noise are two key characteristics of the circadian clock. These features allow the circadian pacemaker to maintain a steady oscillation in a wide range of environmental conditions. The presence of a time-delayed negative feedback loop in the regulatory network generates autonomous circadian oscillations in eukaryotic systems. In comparison, the circadian clock of cyanobacteria is controlled by a strong positive feedback loop. Positive feedback loops with substrate depletion can also generate oscillations, inspiring other circadian clock models. What makes a circadian oscillatory network robust to extrinsic noise is unclear. We investigated four basic circadian oscillators with negative, positive, and combinations of positive and negative feedback loops to explore network features necessary for circadian clock resilience. We discovered that the negative feedback loop system performs the best in compensating temperature changes. We also show that a positive feedback loop can reduce extrinsic noise in periods of circadian oscillators, while intrinsic noise is reduced by negative feedback loops.

## Introduction

Oscillations are everywhere, both in the physical and biological domains. Essential biological processes such as cell cycles^1^, pacemaker cell’s response^2–4^, circadian rhythms^5–7^, calcium oscillations^8^, transcription factor responses^9–13^, hormone secretion^14^, fertility cycles^15^ are only a few examples of biological oscillations^16^. In all of these systems the emergence of oscillations results from a delayed negative feedback loop (NFB)^17^. Based on a study by Novak et al.^18^, nonlinear reaction kinetics, and opposing chemical processes with correct balanced timeframes are also required to create oscillations.

Among different types of biological oscillations, circadian oscillation is widely spread, as the internal clock of most organisms gets synchronized to the environmental day/night cycle changes. Nearly all living organisms maintain an internal clock with a free-running period close to 24 h^19^. Even in the presence of metabolic fluctuations, the circadian clock functions as a precise biological timekeeper^20^. An examination of the nitrogen-fixation property of a cyanobacterium *Synechococcus sp*. provided the first solid evidence for circadian rhythms in prokaryotes^21^. Genetic studies in the fruit fly *Drosophila melanogaster*^6^ and the model filamentous fungus, *Neurospora crassa*^22^, which were later extended to mammals^23^, revealed that circadian oscillations are formed at the molecular level. The core clock genes in mammals (*Per1, Per2, Per3, Cry1, Cry2, Clock, Bmal1, Rev-erbα*, and *Rorα*) cause the rhythmic gene expression and govern the physiological features of circadian rhythms^24,25^. The 2017 Nobel Prize in Physiology or Medicine was awarded to three scientists (Jeffrey Hall, Michael Rosbash, and Michael Young) who identified the working principles of circadian clocks^26^. One of the most well-known fundamental aspects of circadian period homeostasis is temperature compensation^27,28^, which is the maintenance of relatively constant period at varying temperatures. Another unique feature of a circadian oscillator is its robustness against random fluctuations^29,30^. The network motifs or detailed mechanisms that determine temperature compensation and robustness of circadian rhythms remain largely unknown.

Detailed mathematical models have been proposed to explain the behavior of the circadian oscillator. Some of the models contain a relatively small number of species and interactions to generate sustained oscillations. Goodwin developed the simplest model for limit cycle oscillations caused by negative feedback on gene expression^31–33^. Further studies suggest that the presence of positive feedback (PFB) in a circadian clock controls the regulation of the peak concentration of proteins^34^. Combination of a PFB with substrate depletion can also cause sustained oscillation^35^. Later advances have revealed that the presence of PFB improves the robustness of circadian oscillation. PFB induces oscillations in NFB loops without requiring a high degree of cooperativity coefficient (a Hill coefficient of at least 8)^36^. This Hill coefficient can also be reduced by introducing more variables in a NFB model. Based on the underlying mechanism, positive feedback may be divided into three categories: self-activation, Michaelis–Menten degradation, and cross-activation^37^. Ferrell et al. implied that a circadian oscillatory network with interlinked positive and negative feedback is more suited for creating oscillations with configurable frequency and constant amplitude^1^.

Several earlier and recent research^1,38–40^ have contributed to an understanding of the molecular regulatory processes driving biological oscillations, as well as their beneficial qualities such as robustness, tunability, entrainment, and temperature compensation^41^. The Arrhenius law^42^ states that reaction rates increase with temperature^43,44^. As a result, it is expected that oscillation periods would shorten as temperatures rise^45^. In contrast, the circadian clock is temperature compensated and the period is relatively independent of the temperature within a physiological range^45–47^. The primary goal of this paper is to compare key features of basic circadian oscillatory networks in order to determine network motifs that are crucial for the robustness and temperature compensation of oscillation periods.

## Results

**Fig. 1:**
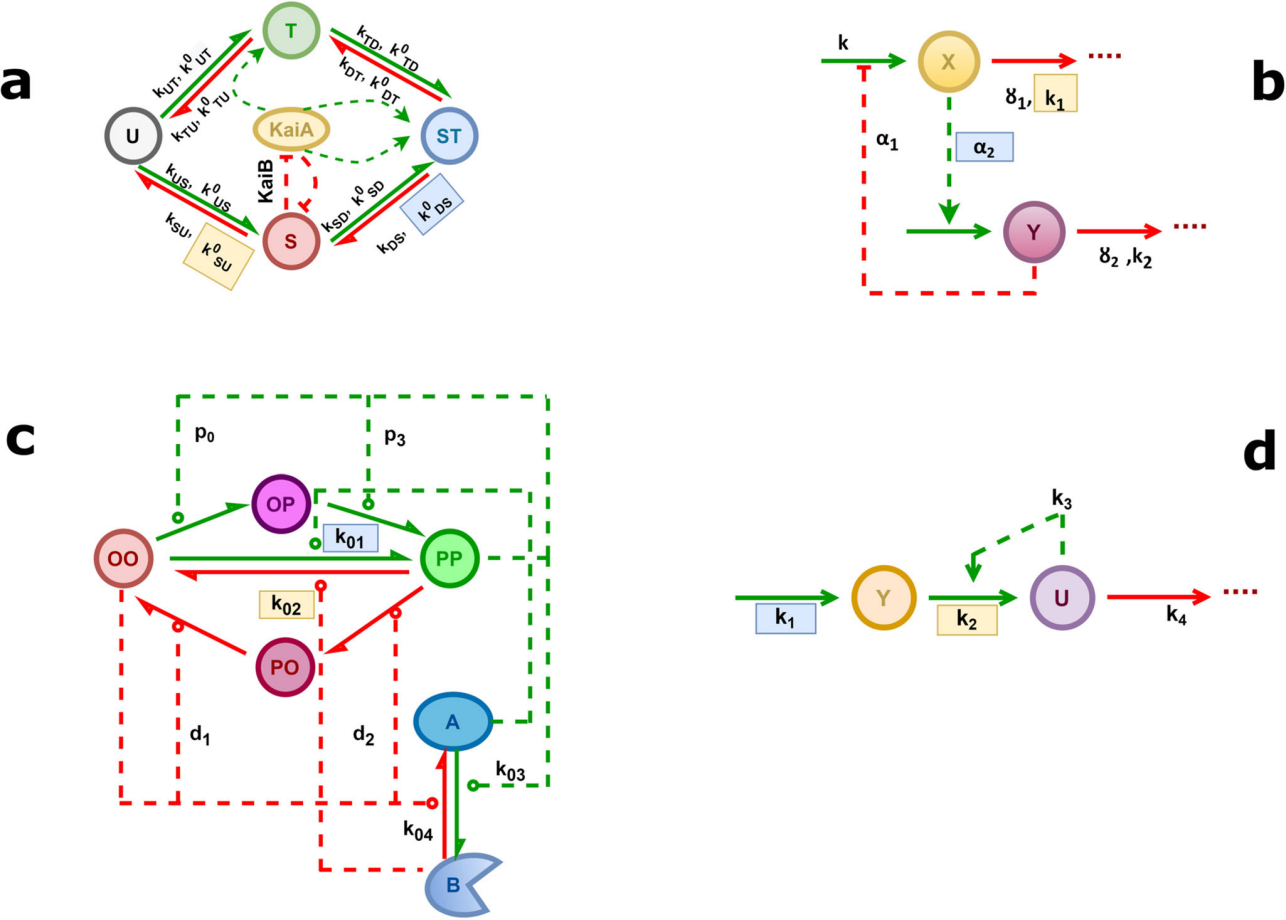
Negative and positive feedback driven oscillatory networks. Schematic diagram of Rust’s cyanobacterial oscillatory network in KaiABC (cyano-KaiABC) which operates through several positive feedback loops (**a**); Goodwin’s negative feedback loop operating between two species (Two-Variable-Goodwin-NFB) (**b**); a single molecule with combined positive and negative feedback that moves through four chemically modified states while interacting with an external molecule having two states (A and B) (cPNFB) (**c**) and Selkov’s substrate depletion oscillatory network, which is the simplest positive feedback loop and substrate depletion driven oscillator (Selkov-PFB) (**d**). activation/phosphorylation and inhibition/dephosphorylation reactions are represented by the green and red arrows, respectively. Inhibition processes are shown by blunt-headed arrows, whereas activation processes are represented by pointed-headed arrows. The reversible reactions are denoted by the double arrows in both directions. The direct reactions (synthesis/degradation, phosphorylation/dephosphorylation) are represented with solid arrows whereas the regulatory indirect reactions (activation/inhibition) are represented by the dashed arrows. Blue shaded parameters are fixed in models reported in Fig. 4 and yellow shaded ones are further fixed in the analysis at Fig. 5.

We present four, greatly divergent, minimalistic models of circadian oscillations and compare them, to gain a better understanding of the key dynamical features of circadian rhythms. Based on the literature^48,49^, most biological clocks^50^ rely on transcriptional-translational negative feedback loops (TTFL). In contrast, the cyanobacterial circadian rhythm^24^ relies on post-translational changes of a single protein species, making the clock a post-translational oscillator (PTO), which is controlled by a positive feedback system^48,51^. This PTO is driven by three clock proteins, KaiA, KaiB, and KaiC, of *Synechococcus elongatus*. The cyanobacteria circadian clock also demonstrates temperature compensated oscillations in an in vitro system^52^. In this experiment, KaiA, KaiB, KaiC, and adenosine triphosphate (ATP) were mixed together to generate sustained oscillation. In vivo, other proteins also bind to distinct forms of KaiC during the day or night. KaiC is a hexamer subunit, and it contains two phosphorylation sites (T432 and S431; abbreviated as T and S). In 24 h, the states change like – U (doubly unphosphorylated state) → T (S/pT, single phosphorylated state) → ST (pS/pT, doubly phosphorylated state) → S (pS/T, single phosphorylated state) → U (where p stands for the phosphorylated site) through phosphorylation and dephosphorylation process^21^. During the daytime, KaiC autophosphorylates, and during the night KaiC dephosphorylates through the same active site. According to Rust et al.^53^, the auto kinase activity of KaiC is increased due to the presence of KaiA, whereas the auto phosphatase activity is enhanced by KaiB^54,55^. Kinetic and biochemical evidence show that one of these phospho forms limits KaiA activity via interacting with KaiB, providing the critical feedback that sustains circadian oscillations^53^. We consider the complex model by Rust et al., which contains several positive feedback loops, and denote this network in the rest of the text as cyano-KaiABC (Fig. 1a).

We also study the ‘conservative Goodwin oscillator,’ a simple and well-studied version of a two-component^17^ negative feedback loop motif (Fig. 1b), which is a simplified version of the TTFL^56^, for this theoretical analysis. In this specific Two-Variable-Goodwin-NFB model, mRNA (variable X) is transcribed from a gene and then translated into protein (variable Y). The latter functions as a repressor, inhibiting the production of mRNA. To avoid negative variable concentration, we employed modified two-variable Goodwin model equations with Michaelis–Menten degradation kinetics^17^.

In a recent study by Hernansaiz et al.^57^ introduced an alternative to Rust’s model^53^ that includes all mass action reactions but a mix of positive and negative feedback to expand the notion of positive-plus-negative feedback oscillators while keeping the kinetics simplified. A single molecule in this model passes through four different chemically modified states dependent on the number of modifications it has: none (OO), single (OP/PO), or double (PP). This core regulator interacts with a molecule that has two states (A and B). We termed the oscillatory network as combined positive-plus-negative feedback (cPNFB) (Fig. 1c).

For considering a positive feedback induced oscillator, we adopt Selkov’s substrate depletion system. We termed the network as Selkov-PFB (Fig. 1d). Because of the presence of substrate inhibition and product activation reactions, this network depicts a simplified kinetic model of an open mono-substrate enzyme (phosphofructokinase) that creates self-oscillations in glycolysis^35^. We include this in our analysis of circadian clock models to consider an oscillator that relies solely on positive feedback and lacks a direct negative feedback loop. Still, in this case we also see that while Y affects U positively, U affects Y negatively (by turning it to U), which leads to the expected opposite sign cross effects, necessary to induce oscillations.

In our investigation, we compared these four minimalistic circadian oscillatory networks. The dynamical mathematical equations of these networks, containing terms for the temperature dependence of each parameter, are given in the methods section. The Time course plot for these oscillatory networks at 298 K temperature is shown in Supplementary Fig. 1. The period of oscillation at 298 K for all the models is 24 h. In terms of regulatory network design, this will allow us to better understand the effects of the key elements of the circadian clock, such as temperature compensation and robustness.

### Robustness analysis

**Fig. 2:**
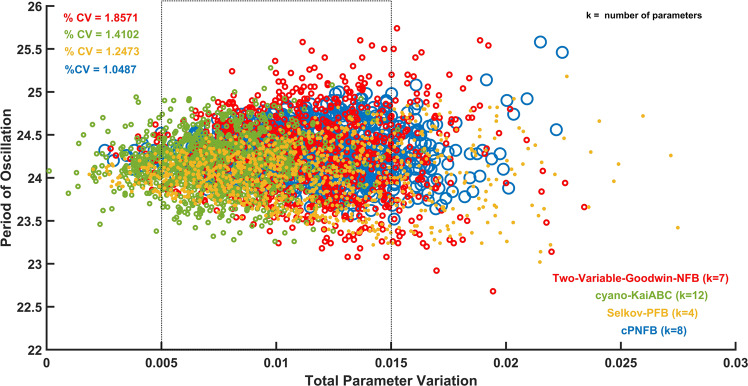
Period of oscillation w.r.t total parameter variations for the four different kinds of oscillatory networks. The graph depicts how the period of oscillations changes in relation to total parameter alterations (Supplementary Note 2A) for the four distinct oscillatory networks shown in Fig. 1. At 298 K for 1000 randomly sampled parameter combinations, the four separate networks are shown in different colors. The inset in the top left lists the percentage of coefficient of variation (% CV) with corresponding colors for all models for 200 sampled parameter sets in each between 0.005 < Total parameter variation < 0.015 (indicated by dashed rectangle box). Parameters are indicated in the supporting information in Supplementary Table 1.

The four investigated models require special parameter combinations to create oscillations with 24 h period (Supplementary Table 1). From a physiological perspective, it is crucial to maintain this period in a noisy chemical environment. We test how the periods of oscillations respond to changes in parameters in the four investigated models. Following earlier ideas on measuring the robustness of biological networks^58,59^, we generated 1000 random parameter combinations from a log-normal distribution of all parameters of the models to measure the robustness of biological systems against the extrinsic fluctuations in parameters (randomly chosen from a log-normal distribution for a multiplicative factor with means equals 1 and standard deviation 0.0142 for all parameters, Supplementary Note 1 for details). We calculated the total parameter variation following earlier works^58,59^, essentially, calculated the arithmetic mean (Supplementary Note 2A) of the reaction rates and displayed it against the periods of oscillations (Fig. 2). This helps to quantify the noise in individual oscillatory networks. The plot shows that the Two-Variable-Goodwin-NFB model has a wider distribution while the cPNFB model has a narrower distribution in terms of the period of oscillations.

For a better quantitative comparison of the extrinsic noise tolerance of the networks, we computed the percentage of coefficient of variation (% CV) of oscillation periods in a narrower range of parameter variations. The percentage of coefficient of variation (% CV) indicates that the Two-Variable-Goodwin-NFB model is the noisiest (% CV = 1.8571). In other words, the Two-Variable-Goodwin-NFB model is the least robust model while the combined positive-plus-negative feedback (cPNFB) model is the most robust. Furthermore, we extended the investigation of the period of oscillation with regards to total parameter fluctuation to seven different temperatures ranging from 283 to 313 K (Supplementary Fig. 2a). We chose a wide range of temperatures as our analysis does not focus on any particular organism. For instance, cyanobacteria can tolerate a large range of temperatures^60–62^. In Supplementary Fig. 2b we present the relationship of the percentage of coefficient of variation (% CV) of the oscillation period changes to the temperature changes in the four different oscillatory networks (Fig. 1).

At all temperatures, we see that the cPNFB model has the smallest variation of periods, while the Two-Variable-Goodwin-NFB model has the largest change with respect to the periods of oscillations. This is true for parameter variations at all temperatures between 283 and 313 K. Only the robustness of the cyano-KaiABC model shows a major temperature dependence, while others seem to have a quite temperature independent robustness (Supplementary Fig. 2b).

The Selkov-PFB model (with the least number of parameters—i.e., four) and the cyano-KaiABC model with 12 parameters show similar distributions despite having different numbers of parameters. We wondered how the number of parameters affect the noise canceling capabilities of models. Bayesian Information Criterion (BIC) is widely used to compare models with altering parameter numbers. It is mostly used to identify which model gives the best fit to an observation, while also considering the number of fitted parameters in the compared models. Thus, BIC is a method that penalizes more complex models by imposing a penalty dependent on the number of parameters evaluated in the model. Following this idea, we examine the correlation between BIC of matching the desired 24 h period of oscillations by the various models (Supplementary Note 2B) at different temperatures (Supplementary Fig. 3). We notice that the Two-Variable-Goodwin-NFB model is still the noisiest at most temperatures (shows the largest BIC), irrespective of its size difference from others. The small Selkov-PFB and the cPNFB models show the lowest noise to parameter changes in this analysis. Thus, we conclude that the number of parameters has no significant impact on the robustness assessment, but it seems the presence of positive feedback in the network leads to lower noise (Supplementary Fig. 3). This is in contradiction with textbook claims on the role of positive and negative feedback in noise increase and decrease, respectively^63^. Here we found the Two-Variable-Goodwin-NFB model as the least robust, despite it is known that negative feedback can serve as a noise reducing motif^64^. In the next steps we will test if negative feedbacks can help temperature compensation of circadian clock models.

### Temperature compensation analysis

When checking the temperature dependence of robustness (Supplementary Fig. 2), we can already notice that the models’ average period is changing with temperature changes, but the level of this change is different in the four models. In order to estimate the temperature compensation properties of these biological oscillators, we have measured their temperature coefficient (*Q*_10_) values^65,66^. In terms of period length changes, we simplify the computation as follows^65^–

1 $${{{Q}}}_{10} = \frac{{{{{\rm{Period}}}}\,{{{\rm{of}}}}\,{{{\rm{oscillation}}}}\,{{{\rm{at}}}}\,{{{\rm{temperature}}}}\,{{{T}}}\,({{{\rm{in}}}}\,{{{\rm{K}}}})}}{{{{{\rm{Period}}}}\,{{{\rm{of}}}}\,{{{\rm{oscillation}}}}\,{{{\rm{at}}}}\,{{{\rm{temperature}}}}\,({{{T}}} + 10)({{{\rm{in}}}}\,{{{\rm{K}}}})}}$$

**Fig. 3:**
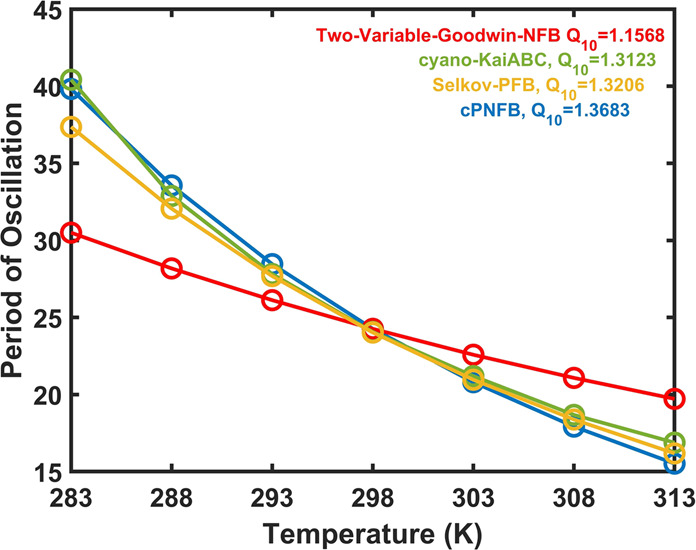
Dependence of oscillation periods on temperature. For all four networks (Fig. 1), the period of oscillation reduces as temperature rises. In the table inset on the top right, the *Q*_10_ values between 293 and 303 K for each model are also presented. Parameters are listed in the supporting information in Supplementary Table 1. The lowest *Q*_10_ value in Fig. 3 suggests that the Two-Variable-Goodwin-NFB model is better temperature compensated than the other three models. The trend also shows that when the temperature rises, the duration of the period decreases for all four models. Two-Variable-Goodwin-NFB performs well in temperature compensation but poorly in robustness (Fig. 2). As all the models are imperfectly temperature compensated, further assumptions might need to be tested.

Because circadian oscillators should maintain their approximately 24 h period, they need to be temperature compensated. We plotted how the periods of oscillations respond to temperature changes in all four models (Fig. 3). We can observe that all of these models perform some temperature compensation, we still observe a great effect of temperature changes on the periods of oscillations.

### Individual temperature compensated reactions

**Fig. 4:**
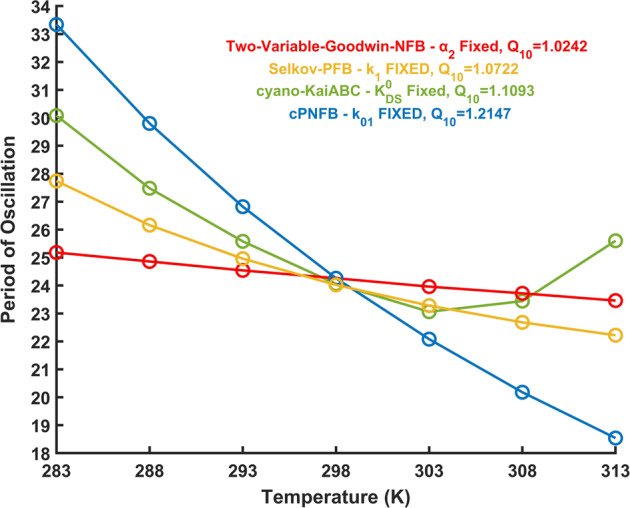
Dependence of oscillation periods on temperature if a single reaction rate is temperature independent. The figure indicates how far the period of oscillations varies with temperature in all four investigated oscillatory network motifs (Fig. 1), when the rate of a single reaction is fixed (labeled in legend), but all others are allowed to respond to temperature changes. Parameters are indicated in the supporting information in Supplementary Table 1. Additionally, each model’s *Q*_10_ values between 293 and 303 K are shown in the figure. The fixed parameters are shown in the shaded blue color boxes in Fig. 1.

Instead of expecting that the period of oscillations is temperature compensated by a compensatory effect of reaction rate responses to temperature change^67^, we can consider that an individual reaction may be temperature compensated, which have a major effect in compensating period changes^27,44,68^. Following ideas by Hong et al.^69^ and observations on temperature insensitivities of key circadian clock reaction^70,71^, we have checked what happens if we fix the rates of a single reaction while allowing all others to respond to temperature change? The goal of this research is to identify the reactions which could be responsible for temperature compensation in the various oscillatory networks. We explored how far the period of oscillations varies with temperature in a pure NFB (Supplementary Fig. 4b) and pure PFB motif (Supplementary Fig. 4d) when a single rate is constant, and we also checked the same in complex oscillatory networks with several combinations of positive and negative feedback loops (Supplementary Fig. 4a, Supplementary Fig. 4c) as well. Figure 4 depicts the best performing models, where a single rate was assumed to be temperature independent, while all others respond to temperature changes.

It can be concluded that the Two-Variable-Goodwin-NFB model outperforms the others (Fig. 4) when the *α*_2_ reaction rate (the rate at which the activator *X* triggers the synthesis of its inhibitor, *Y*, blue background on Fig. 1b) of the model is temperature resistant. The other models also improved on their *Q*_10_ values but are still quite sensitive to temperature changes. Interestingly Rust’s cyanobacterial circadian clock model, with rate $k_{\rm{DS}}^0$ fixed (the rate at which the doubly phosphorylated state ‘*ST*’ transitions to the singly phosphorylated state ‘*S*’, blue background on Fig. 1a), show a temperature dependence curve with an increase in periods at larger temperatures.

**Fig. 5:**
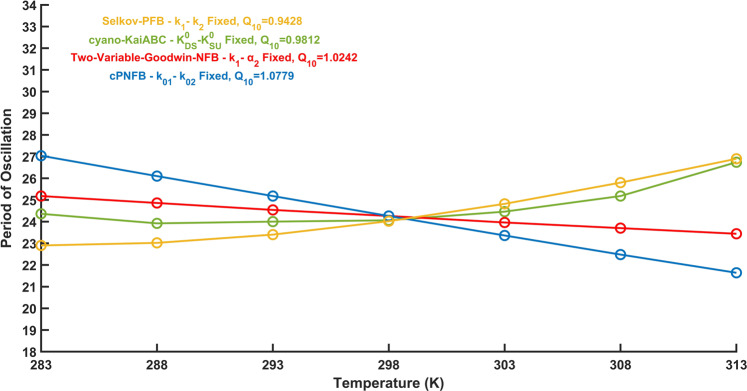
Dependence of oscillation periods on temperature if two reactions are temperature change resistant. The image shows how far the period of oscillations varies with temperature in all four oscillatory network motifs (Fig. 1) when the rates of two reactions are fixed simultaneously (indicated in legend) while all others are permitted to respond to temperature change. We only plot here the parameter combinations, which produce the lowest *Q*_10_ values. Parameters are shown in the supporting information in Supplementary Table 1. The second fixed parameters along with the primary fixed parameters (Fig. 4) are described in Fig. 1 with yellow and blue shaded boxes respectively.

If we consider temperature independence of two reaction rates, then we observe strong temperature compensation of the periods of the oscillators (Fig. 5). The Two-Variable-Goodwin-NFB (plotted in red color) and cPNFB (plotted in blue color) exhibit the predicted decrease in period for temperature increase (Fig. 5). On the other hand, the Selkov-PFB network (plotted in yellow) contains only four parameters, and it seems, if we fix two of those, the other two could be quite well temperature compensated, but the period increases as a function of temperature. The most complicated model in our analysis is the cyano-KaiABC model (shown in green), which has a complex temperature response what shows a minimum around 24 h. The cyano-KaiABC model works well between 283 and 298 K temperatures, however, the period of oscillation begins to differ significantly from 24 h at 303 K.

Supplementary Figs. 5–8 show all possible combinations of two temperature compensated parameters for the Two-Variable-Goodwin-NFB, cyano-KaiABC, cPNFB, and Selkov-PFB motifs, respectively. By comparing Figs. 4 and 5, we can deduce that Two-Variable-Goodwin-NFB with a single temperature compensated parameter (*α*_2_) performs almost identically to the two-parameter fixed scenario (*k*_1_-*α*_2_), while the other three networks show further improvements for the fixation of a second rate (these rates are labeled by blue and yellow on Fig. 1). It can be observed that the *α*_2_ rate of the Two-Variable-Goodwin-NFB network is directly controlling the negative feedback loop. The reaction rates, which should be temperature independent in the cyano-KaiABC and cPNFB models are also controllers of negative feedback loops. There is no negative feedback loop in the Selkov-PFB, there the most crucial parameter seems to be the one that controls the synthesis of the substrate of the positive feedback loop. Still in all models we see some improvements on temperature compensation with the fixation of a second reaction rate. The reduced values of *Q*_10_ in Fig. 5 make the periods of oscillations better temperature compensated than shown in Fig. 4, where only one rate was fixed in each model.

### Separating the effects of positive and negative feedbacks

**Fig. 6:**
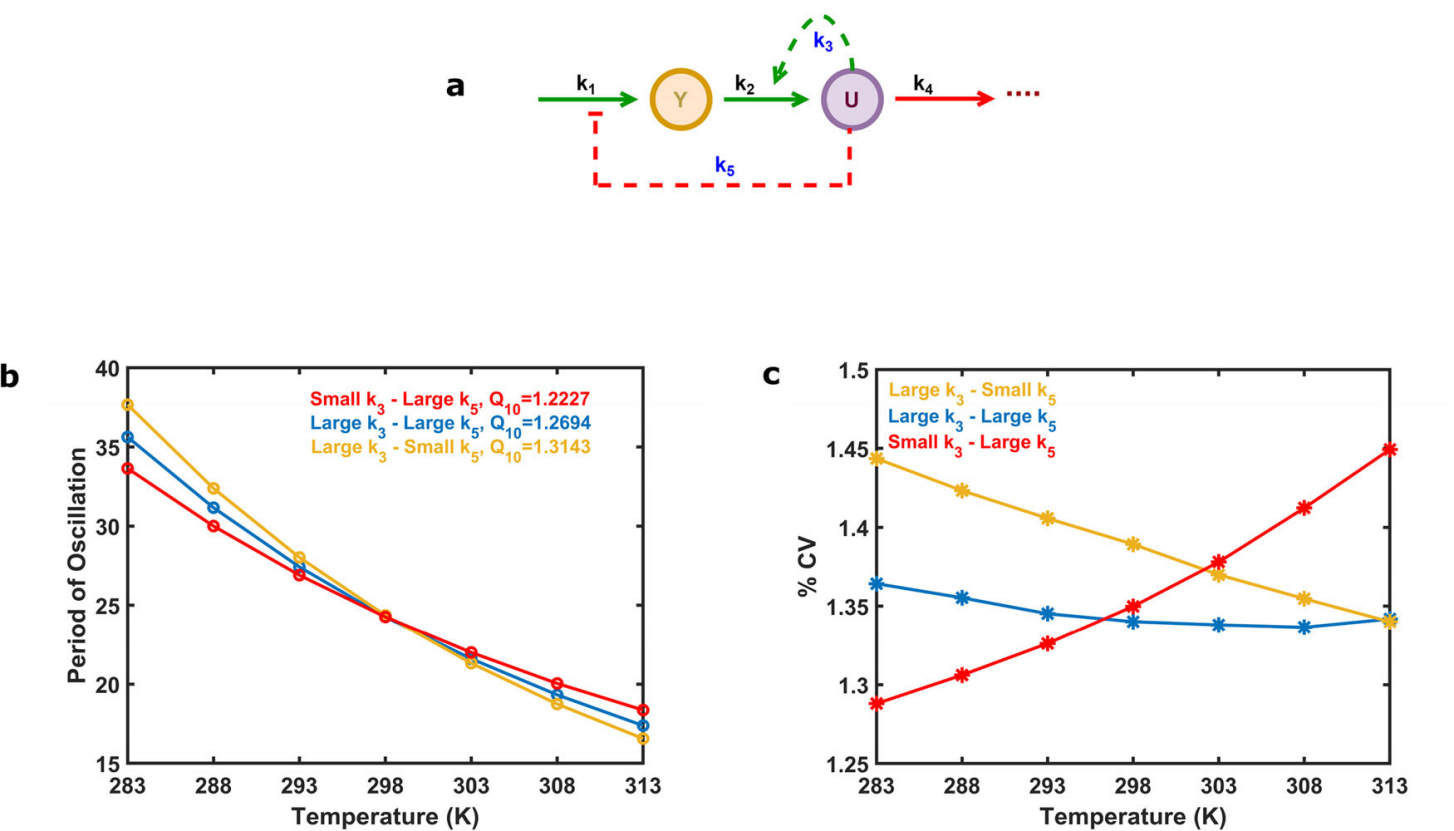
Selkov-like positive feedback oscillator with an additional negative feedback loop. The wiring diagram of the oscillatory network (**a**). The inherent autocatalytic PFB in a Selkov model is indicated by the dashed green arrowhead (*k*_3_) and the external additional NFB is denoted by dashed red line with blunt head (*k*_5_). *k*_3_ and *k*_5_ rates are highlighted in blue color as these were altered. **b** Period of oscillation changes with temperature for the strong NFB – weak PFB (red), weak NFB – strong PFB (yellow) and strong NFB – strong PFB (blue) cases. **c** Robustness, measured as the percentage of coefficient of variations (%CV) at various temperatures for the three different cases explained on panel b in the presence of extrinsic noise. Parameters are listed in the Supplementary Table 1E.

The four above investigated networks have different kinetics and complexity (Fig. 1), in order to have a systematic approach to study the role of positive and negative feedbacks in the robustness and temperature compensation of circadian clock models, we have extended our analysis with a Selkov-like positive feedback-based model with an additional negative feedback loop (Fig. 6a). In order to ensure the compared models have similar sizes and kinetics, we keep the size of the network the same and control the strength of a single rate for the positive (*k*_3_) and the negative feedback loop (*k*_5_) (parameter values are indicated in the Supplementary Table 1E, and rate constants at 298 K are displayed in the Supplementary Table 1E). The temperature compensation analysis of the combined model (Fig. 6b) suggests that strong NFB with weak PFB (small *k*_3_-large *k*_5_ case, with red line) leads to better temperature compensation (lowest *Q*_10_ values) than other combinations (Fig. 6b). This matches with our above findings (Fig. 3) that negative feedback improves temperature compensation. A robustness analysis (Fig. 6c) in the presence of extrinsic noise^72^ shows that robustness of networks has a strong temperature dependence. Combination of positive and negative feedbacks (blue) shows the least temperature dependence on noise, while strong PFB alone (large *k*_3_-small *k*_5_ case, with yellow line) is less robust. An interesting observation is that strong NFB (small *k*_3_-large *k*_5_ case, with red line) becomes less robust (Fig. 6c) at higher temperatures. This is probably because at higher temperatures the faster reaction rates reduce the delays of feedback loops, which is one of the key components for autonomous oscillations and period determination.

**Fig. 7:**
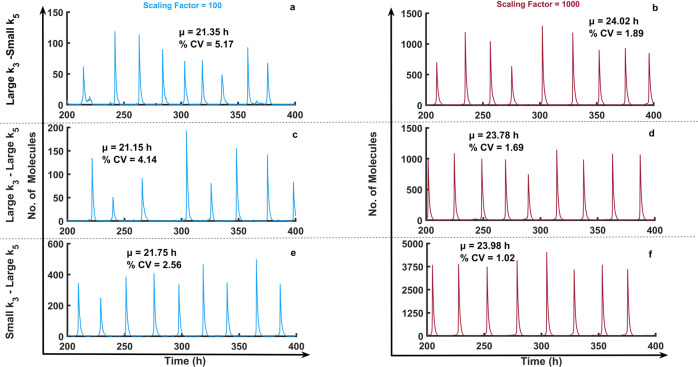
Effects of intrinsic noise on the Selkov-like positive feedback oscillator with an additional negative feedback loop. (**a**, **c**, **e**) Time course trajectory samples of species *U* (Fig. 6a) with the volume related scaling factor of the Gillespie simulations set to 100 and (**b**, **d**, **f**) or 1000 at 298 K. Percentage of coefficient of variations (% CV) and mean period (μ) at 298 K were calculated for 100 cycles and reported on top of each panel Model is reported in the “Methods” section. Parameters are listed in Supplementary Table 1E.

The observation that negative feedback might increase noise contradicts earlier findings, which showed noise reducing capabilities of negative feedback loops^64^. Specifically, previous work indicated that negative feedback loops could reduce cell-cell variation due to intrinsic noises^64,72^. To investigate potential roles of negative and positive feedback loops with respect to noise, we performed stochastic simulations of the combined Selkov-like positive-negative feedback model (Fig. 6a). Stochastic simulations of this system at various low molecular abundance levels reveal that at 298 K, strong NFB with weak PFB (small *k*_3_-large *k*_5_ case) results in the most robust against intrinsic noise, caused by low molecular abundances (Fig. 7).

## Discussion

The mechanisms for temperature compensation of biological oscillators remain an unsolved puzzle. We have shown here that robustness and temperature compensation of circadian oscillators come at the cost of one another. It is clear from Fig. 2 and Fig. 3 that Two-Variable-Goodwin-NFB is the least robust for parameter fluctuations (possesses a high % CV value for period of oscillations) while it is better temperature compensated (lower *Q*_10_ value) compared to the others. On the other end of the scales, the cPNFB circadian oscillatory network is the most robust and least temperature compensated (Fig. 2 and Supplementary Figs. 2, 3). These are two extreme examples of robustness and temperature compensation in the four models that we interrogated. Based on these results and on the systematic analysis of a combined positive plus negative feedback systems (Fig. 6) we can conclude that pure NFB cannot reduce noise in the system efficiently (Two-Variable-Goodwin-NFB), whereas the presence of a PFB in the network increases robustness of the system (at high temperatures) without reducing much on the temperature compensating capabilities of negative feedback loops in the presence of extrinsic noise (Fig. 6). In contrast, a NFB increases the robustness of the system in the presence of intrinsic noise (Fig. 7).

The cyano-KaiABC model of Rust et al.^53^ is a quite complex model, containing both positive and negative feedbacks and highly nonlinear kinetics. This model shows good temperature compensation when $k_{\rm{DS}}^0$ and $k_{\rm{SU}}^0$ are both fixed (Fig. 5), but it is not the most robust or best temperature compensated model even though it has the highest number of parameters (*k* = 12). Furthermore, we believe that the linear mass action kinetics in the cPNFB circadian oscillatory network might be the distinguishing factor for its least noisy behavior when compared to the other models, where nonlinear terms might exaggerate any noise in the system (“Methods” section). Mass action kinetics provides precision even at low input signal values, whereas nonlinear kinetics such as Hill or Michaelis–Menten lack precision due to overly complicated combinations and scaling factors of different reaction rates used to define the kinetics^73^.

Minimal models of the plant circadian clock with various levels of complexity were also tested for temperature compensation by Avello et al.^74^. Our systematic analysis revealed that negative feedback loops are needed for good temperature compensation and the combination of positive and negative feedback loops can lead to lower noise in circadian clocks. We also see that temperature independence of reactions, which are crucial for negative feedback loops can help the whole systems to drive temperature compensated oscillations. These results could explain why in higher eukaryotes the classical delayed negative feedback loop-based system drives circadian clock, while in the cyanobacteria in vitro circadian oscillator we see a positive feedback-based KaiC system, where the temperature compensation is achieved through temperature independence of the key reactions in the system^67^. These results also support earlier ideas that positive feedbacks can make circadian clock more robust^1,69^.

Feedback loops can be constructed in many different ways on the molecular level^75^. Feedbacks can drive activation or inhibition steps or affect both. Furthermore, kinetics of reactions could also have an effect of the precise dynamics of oscillators. We have investigated here a wide selection of such systems but systematically tested only a single combination model (Figs. 6 and 7). Thus, our analysis has its limitations and could be further expanded by studying effects of various kinetics, regulatory effects, and basal reaction rates.

Despite these limitations, the proposed model engineering and comparing strategies can be useful for the designs of synthetic circuits, which are desired to act robustly against environmental fluctuations, including temperature changes. This could be relevant to engineering technologies of cyanobacteria species for biomass production, involving light-harvesting^76^. We believe that our study will further support this blooming research field.

## Methods

Here we present the mathematical models (A–D) of the four oscillatory networks (Fig. 1) and an additional oscillatory network described in Fig. 6a (E):

A. cyano-KaiABC network
  2 $$U = {\rm{kaiC}} - T - D - S$$
  Where, *U* = unphosphorylated form; *T* = threonine phosphorylated kaiC; *D* = double phosphorylated kaiC (= *ST*); and *S* = serine phosphorylated kaiC. The dynamical equations and parameter values (Supplementary Table 1A) for this model are taken from the original model of Rust et al.^53^.
  3 $${\rm{kaiA}} = A = {{{\mathrm{max}}}}(0,A - 2 \cdot S)$$
  4 $$k_{ij} = k_{ij}^0 + k_{ij}^A \cdot \frac{A}{{k_{\rm{half}} + A}}$$
  Where, i,j ε {U,T,D,S}.
  5 $$\frac{{dT}}{{dt}} = k_{UT} \cdot U + k_{DT} \cdot ST - k_{TU} \cdot T - k_{TD} \cdot T$$
  6 $$\frac{{dST}}{{dt}} = k_{TD} \cdot T + k_{SD} \cdot ST - k_{DT} \cdot ST - k_{DS} \cdot ST$$
  7 $$\frac{{dS}}{{dt}} = k_{US} \cdot U + k_{DS} \cdot ST - k_{SU} \cdot S - k_{SD} \cdot S$$
B. Two-Variable-Goodwin-NFB network
  The dynamical equations for this model are taken from Gonze et al.^17^. The parameters for this network are changed in such a way (Supplementary Table 1B) that the period of oscillations is 24 hours at 298 K temperature.89
C. cPNFB Network
  The dynamical equations for this model are taken from Hernansaiz et al.^57^. The parameters for this network were changed in such a way that the period of oscillations (Supplementary Table 1C) is 24 h at 298 K temperature.
  10 $$\frac{{dOO}}{{dt}} = k_{02} \cdot B \cdot PP + d_1 \cdot OO \cdot PO - k_{01} \cdot A \cdot OO - p_0 \cdot OO \cdot PP$$
  11 $$\frac{{dPP}}{{dt}} = k_{01} \cdot A \cdot OO + p_3 \cdot OP \cdot PP - k_{02} \cdot B \cdot PP - d_2 \cdot OO \cdot PP$$
  12 $$\frac{{dOP}}{{dt}} = p_0 \cdot OO \cdot PP - p_3 \cdot OP \cdot PP$$
  13 $$\frac{{dPO}}{{dt}} = d_2 \cdot OO \cdot PP - d_1 \cdot OO \cdot PO$$
  14 $$\frac{{dB}}{{dt}} = k_{03} \cdot A \cdot PP - k_{04} \cdot B \cdot OO$$
  15 $$\frac{{dA}}{{dt}} = k_{04} \cdot B \cdot OO - k_{03} \cdot A \cdot PP$$
D. Selkov-PFB Network
  The dynamical equations for this model are modified from the original paper by SEL’KOV^35^. The parameters for this network were changed in such a way (Supplementary Table 1D) that the period of oscillations is 24 h at 298 K temperature.
  16 $$\frac{{dY}}{{dt}} = k_1 - k_2 \cdot Y - k_3 \cdot U^2 \cdot Y$$
  17 $$\frac{{dU}}{{dt}} = k_2 \cdot Y + k_3 \cdot U^2 \cdot Y - k_4 \cdot U$$
E. Selkov-like PFB based model with an additional NFB loop

The mathematical model of Selkov-like PFB based model with an additional NFB loop (Fig. 6a) is presented as:

18 $$\frac{{dY}}{{dt}} = \frac{{k_1}}{{1 + k_5 \cdot U}} - k_2 \cdot Y - k_3 \cdot U^2 \cdot Y$$

19 $$\frac{{dU}}{{dt}} = k_2 \cdot Y + k_3 \cdot U^2 \cdot Y - k_4 \cdot U$$

Here, $k_3$ is the inherent activatory autocatalytic positive-feedback influence on the species *U* in a typical Selkov model (Fig. 1d). *U* exhibits an additional NFB by imposing inhibition to the synthesis of species *Y* with the rate $k_5$. Because of the additional NFB in this model, the total parameter number (Supplementary Table 2) is equal to 5.

The mathematical equation represents above can be expressed in terms of number of molecules by introducing a scaling factor ($V_s$) on the both sides of the equation. With the Gillespie approach^77^, we utilized the following equation for the robustness analysis illustrated in Fig. 7:

20 $$\frac{{dN_Y}}{{dt}} = \frac{{k_1 \cdot V_s^2}}{{V_s + k_5 \cdot N_U}} - k_2 \cdot N_Y - k_3 \cdot \left( {\frac{{N_U}}{{V_s}}} \right)^2\, \cdot \,N_Y$$

21 $$\frac{{dN_U}}{{dt}} = k_2 \cdot N_Y + k_3 \cdot \left( {\frac{{N_U}}{{V_s}}} \right)^2 \,\cdot\, N_Y - k_4 \cdot N_U$$

Here, $N_Y$ and $N_U$ are the number of *Y* and *U* species present in the system.

The calculated reaction rates that apply to low *k*_3_ or *k*_5_ values are roughly one-fifteenth of the larger *k*_*3*_ or *k*_*5*_ values. With a low *k*_3_ value and a large *k*_5_ value, the system will have a dominating NFB (strong NFB – weak PFB), whereas with a large *k*_3_ and a low *k*_5_, the system will have a dominant PFB (weak NFB – strong PFB). When both the rates are large and equal, the system will display the combined effect of a PFB and a NFB (strong NFB – strong PFB).

The parameter values for the three cases shown in Fig. 6b, c and Fig. 7 are indicated in the Supplementary Table 1E.

### Simulation settings

The dynamical equations discussed above have been simulated in Matlab (ver. R2021b) using the ODE45 solver tools. Each reaction rate represented in the ordinary differential equations above, has been rewritten in an Arrhenius equation form, representing the temperature dependence of reaction rates as:

22 $${\rm{rate}} = A_e \cdot e^{\frac{{ - E}}{{R \cdot T}}}$$

where the pre-exponential factor for all rates is, *A*_e_ = 383.83 (arbitrary unit, ‘A.U.’)^27^, and this is the parameter that is randomly chosen for the robustness analysis. The gas constant = *R* = 8.3144598 J K^−1^ mol^−1^ and *T* = temperature is measured in Kelvin.

## Supplementary information

Supplementary Information

## Data Availability

All data used for the simulations are presented in the Supplementary Information.

## Code availability

The Matlab code of simulated models are available at GitHub: https://github.com/SuchanaChakravarty/Systematic-analysis-of-NFB-PFB-loops-for-robustness-temperature-compensation-in-circadian-rhythm.git.
