# Supplementary material for: Systematic analysis of negative and positive feedback loops for robustness and temperature compensation in circadian rhythms
Source: NPJ Syst Biol Appl. 2023 Feb 11;9:5. doi: 10.1038/s41540-023-00268-7 (PMC9922291; doi:10.1038/s41540-023-00268-7)
Supplement: Supplementary file 1 — Supplementary Information [file 41540_2023_268_MOESM1_ESM.pdf]

Supporting Information

Systematic analysis of negative and positive feedback loops for robustness and temperature compensation in circadian rhythms

Suchana Chakravarty[1], Christian I. Hong[2,3] and Attila Csikasz-Nagy[1]

[1] Faculty of Information Technology and Bionics, Pázmány Péter Catholic University, Budapest, Hungary

[2] Department of Pharmacology & Systems Physiology, University of Cincinnati, Cincinnati, OH, USA

[3] Division of Developmental Biology, Cincinnati Children's Hospital Medical Center, Cincinnati, OH, USA

chakravarty.suchana@itk.ppke.hu, christian.hong@uc.edu, csikasz-nagy.attila@itk.ppke.hu

Contents

Supplementary Figure 1. Time-course diagram for the four studied oscillatory networks from Figure 1. 3

Supplementary Figure 2. Robustness analysis of the four investigated oscillatory networks. 4

Supplementary Figure 3. Analysis of Bayesian Information Criterion (BIC) of the four models at various temperatures. 5

Supplementary Figure 4. Dependence of oscillation periods on temperature if a single reaction is temperature independent. 6

Supplementary Figure 5. Periods of oscillations when two rates are temperature independent in the Two-Variable-Goodwin-NFB network. 7

Supplementary Figure 6. Periods of oscillations when two rates are temperature independent in the cyano-KaiABC network. 8

Supplementary Figure 7. Periods of oscillations when two rates are temperature independent in the cPNFB network. 9

Figure 8. Have Periods of oscillations when two rates are temperature independent in the Selkov-PFB network. 9

Supplementary Table 1: Parameters 10

Supplementary Table 1A. Parameters for cyano-KaiABC Network 10

Supplementary Table 1B. Parameters for Two-Variable-Goodwin-NFB Network 11

Supplementary Table 1C. Parameters for cPNFB Network 11

Supplementary Table 1D. Parameters for Selkov-PFB Network 12

Supplementary Table 1E. Selkov-like PFB based model with an additional NFB loop 12

Supplementary Note 1. Addition of noise to the models 13

Supplementary Figure 9. A histogram of multiplicative factor for pre-exponential values chosen at random from a log normal distribution. 14

Supplementary Note 2. Statistical Measurements 14

Supplementary Note 2A. Total Parameter Variation 14

Supplementary Note 2B. Bayesian Information Criterion (BIC): 14

Supplementary Table 2. Total Number of Parameters 15

Supplementary Figure 10. The relationship between total parameter variations and oscillation period for a Selkov-like PFB based model with an additional NFB loop. 16

Supplementary References 17

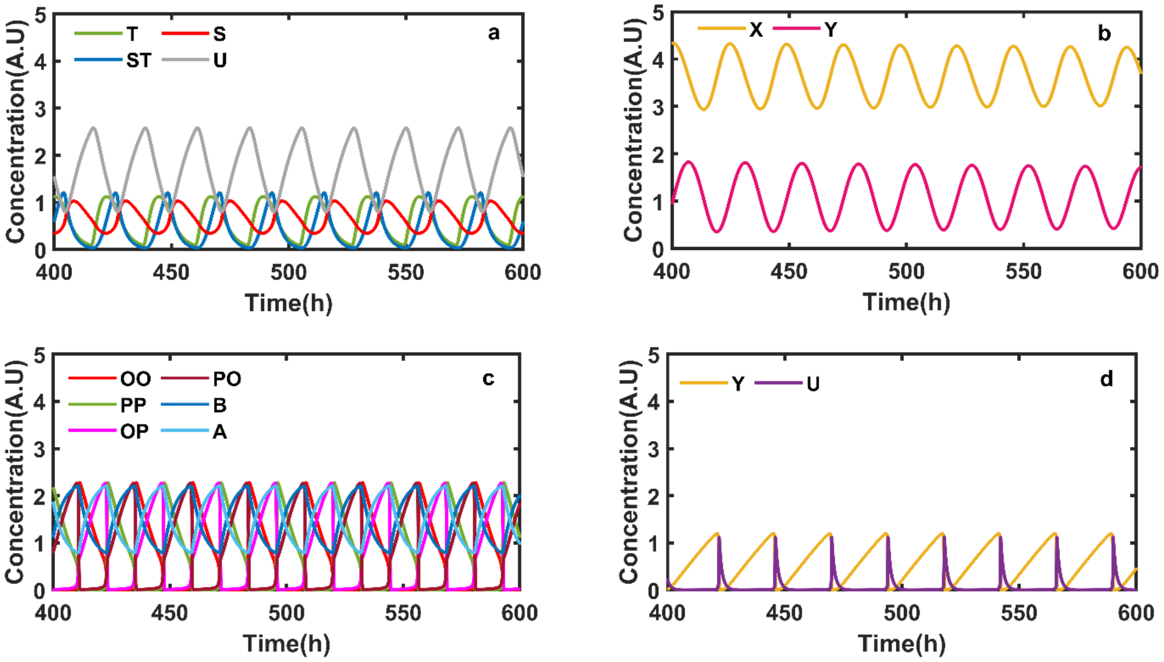

Supplementary Figure 1. Time-course diagram for the four studied oscillatory networks from Figure 1. The figure shows how the concentration of species changes over time for the four oscillators cyano-KaiABC (a); Two-Variable-Goodwin-NFB (b); cPNFB (c) and Selkov-PFB (d). The simulations have been carried out at 298K temperature. Initial concentrations of the molecules and the parameters are listed in Supplementary Table 1.

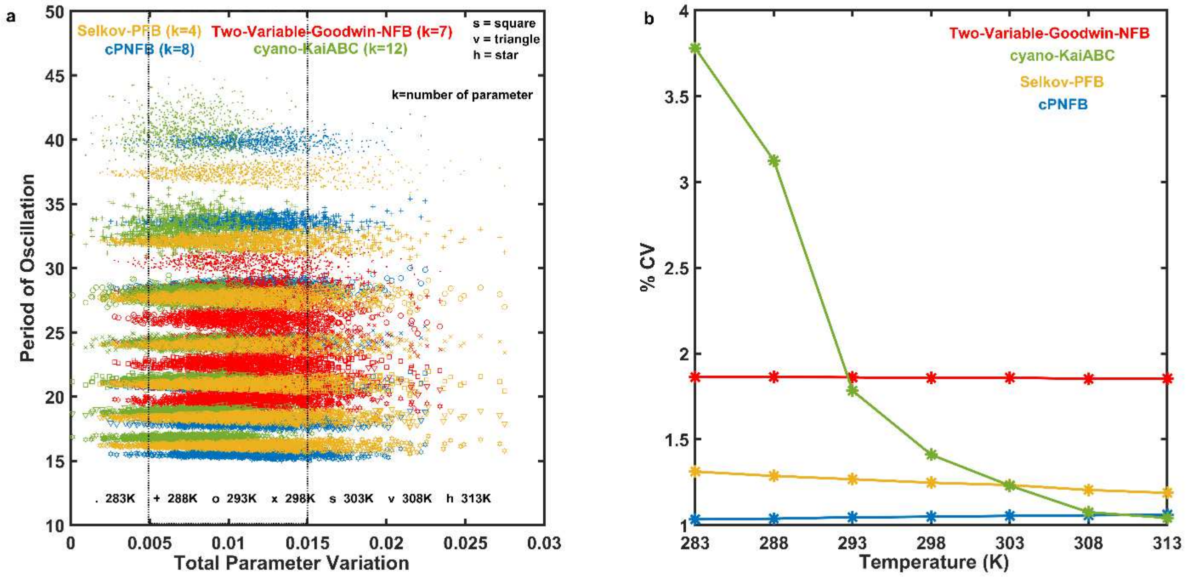

Supplementary Figure 2. Robustness analysis of the four investigated oscillatory networks. The panel (a) shows how the period of oscillations varies in proportion to overall parameter changes (Supplementary Note 2A) for one thousand random sample points at seven different temperatures in four oscillatory networks (Figure 1). These networks are distinguished by four separate colors, and the distinct seven temperatures are marked by different shaped markers. Initial concentrations of the molecules and the parameters are listed in Supplementary Table 1. The panel (b) displays how the percentile co-efficient of variation (% CV, i.e. standard deviation over the mean) changes with temperature for the four oscillatory networks (Figure 1). The percentile co-efficient of variation (% CV) has been calculated from the period of oscillations indicated in Supplementary Figure 2a for 200 sampled parameter sets in each between 0.005 < Total parameter variation < 0.015 (indicated by dashed rectangle box, Supplementary Figure 2a).

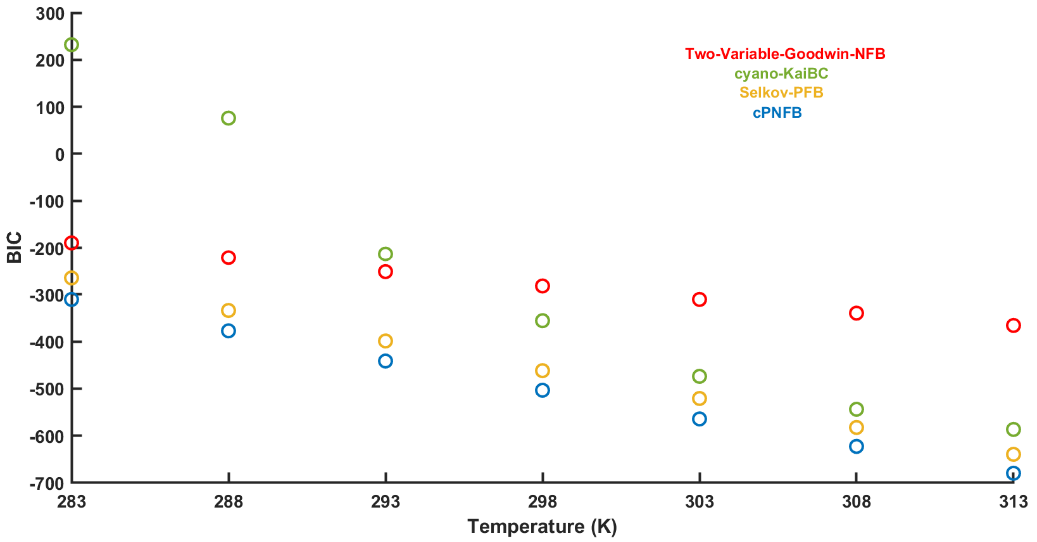

Supplementary Figure 3. Analysis of Bayesian Information Criterion (BIC) of the four models at various temperatures. For each model (Figure 1), the BIC (Supplementary Note 2B) has been displayed against various temperatures. The BIC of the Two-Variable-Goodwin-NFB model is the highest above 298K. The sample size of the analysis is 200 and the individual set corresponds to 0.005 < Total parameter variation < 0.015 (displayed in Figure 2 and Supplementary Figure 2). Initial concentrations of the molecules and the parameters are listed in Supplementary Table 1.

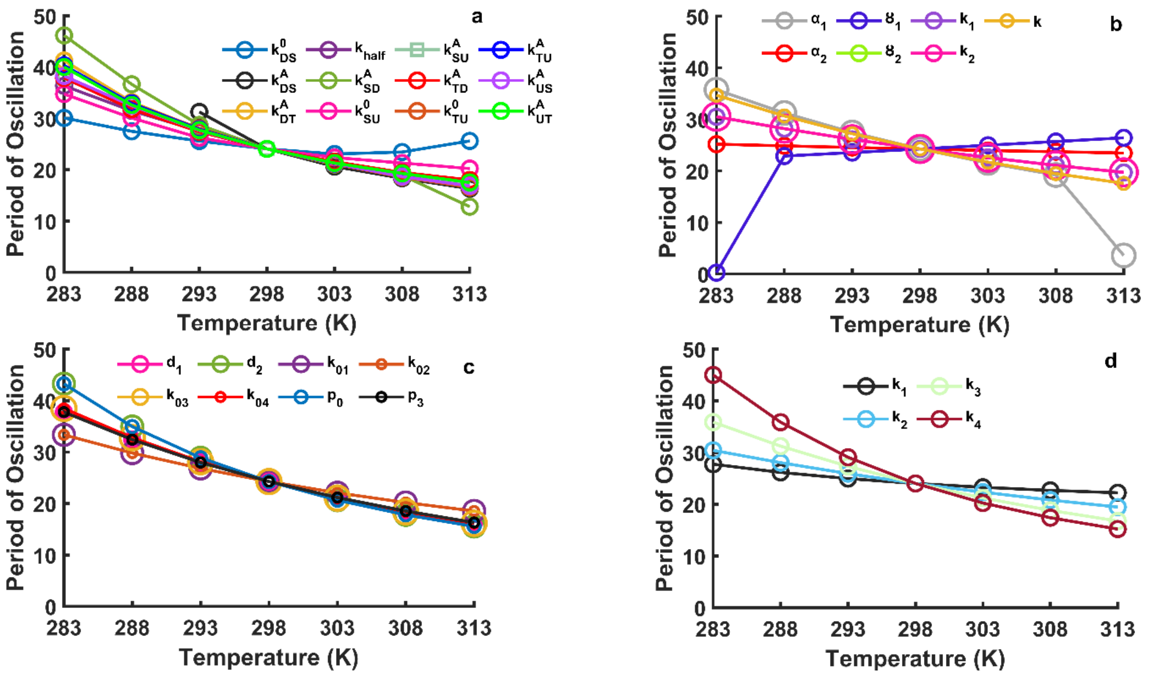

Supplementary Figure 4. Dependence of oscillation periods on temperature if a single reaction is temperature independent. The image shows how much the period of oscillations varies with temperature in all four oscillatory network motifs (Figure 1) when the rate of a single reaction is fixed (designated in the legend) but all others are permitted to respond to temperature changes. The analysis is done for all of the reactions associated with each model. Initial concentrations of the molecules and the parameters are listed in Supplementary Table 1. At the highest and lowest temperatures some models fail to show oscillations, thus periods drop to 0.

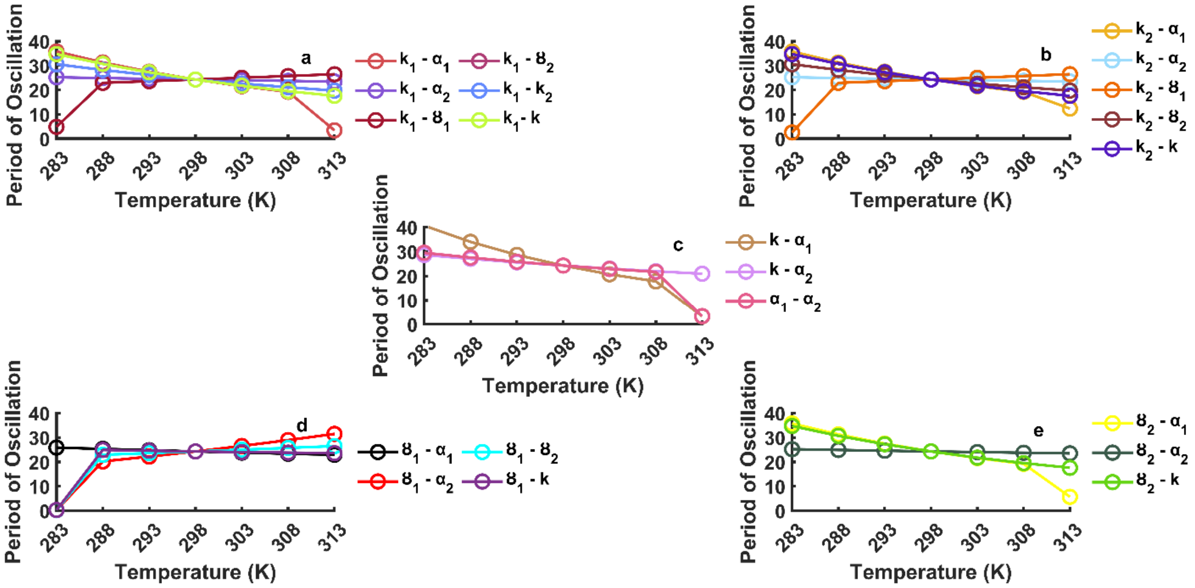

Supplementary Figure 5. Periods of oscillations when two rates are temperature independent in the Two-Variable-Goodwin-NFB network. The plots (a-e) demonstrate how far the periods of oscillations vary with temperature in the Two-Variable-Goodwin-NFB network when the two reaction rates are temperature independent (as specified in the legend) and all others are allowed to respond to temperature change. The analysis is done for all of the possible reaction rate combinations associated with this model. Initial concentrations of the molecules and the parameters are listed in Supplementary Table 1B.

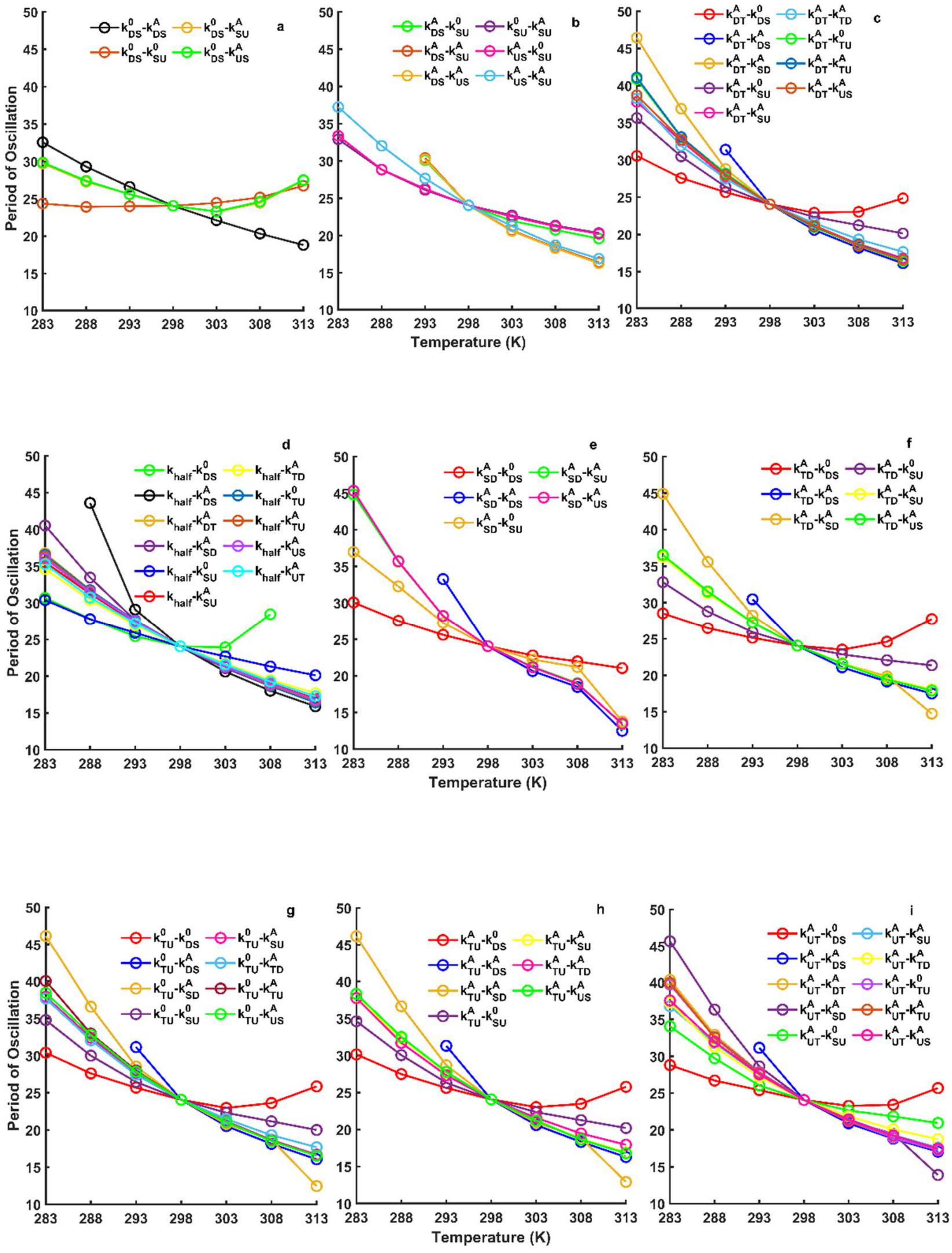

Supplementary Figure 6. Periods of oscillations when two rates are temperature independent in the cyano-KaiABC network. The subplots (a-i) show how the period of oscillations in the cyano-KaiABC network vary with temperature when the two reaction rates are temperature compensated (as described in the legend) and all others are allowed to respond to temperature change. The analysis is done for all of the possible reaction rate combinations associated with this model. Initial concentrations of the molecules and the parameters are listed in Supplementary Table 1A. At the highest and lowest temperatures some models fail to show oscillations, thus periods drop to 0.

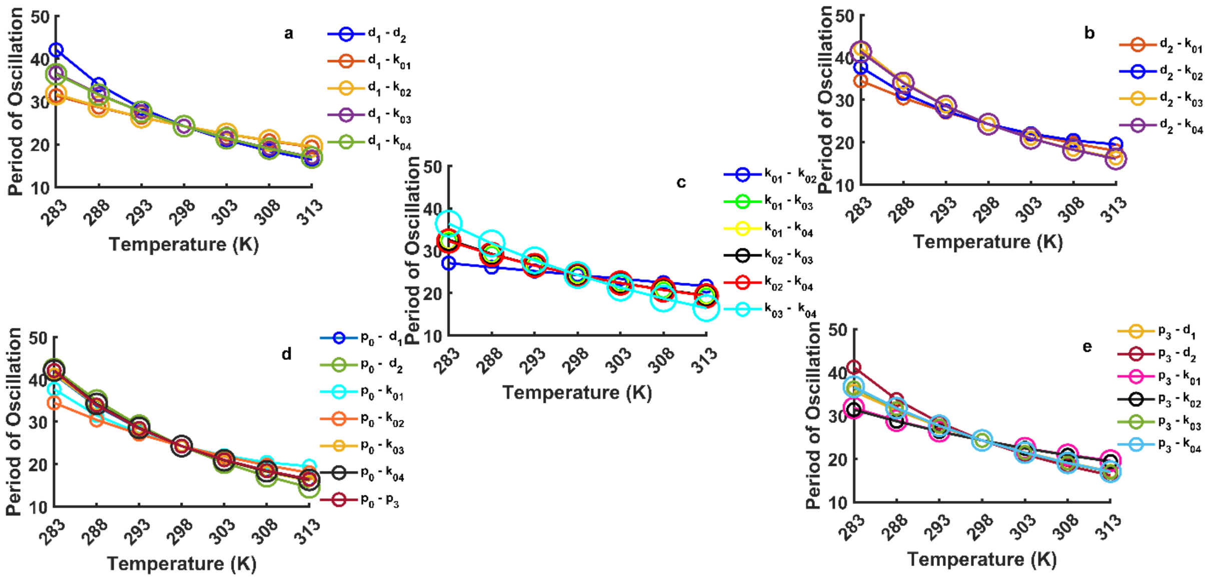

Supplementary Figure 7. Periods of oscillations when two rates are temperature independent in the cPNFB network. The subplots (a-e) show how the period of oscillations varies with temperature in the cPNFB network when the two reaction rates are temperature compensated (as described in the legend) and all others are permitted to respond to temperature change. The analysis is done for all of the possible reaction rate combinations associated with this model. Initial concentrations of the molecules and the parameters are listed in Supplementary Table 1C.

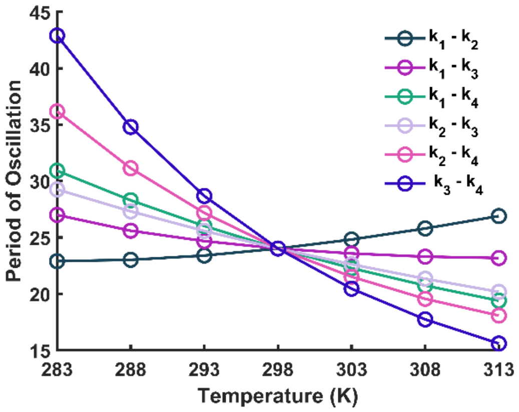

Figure 8. Have Periods of oscillations when two rates are temperature independent in the Selkov-PFB network. The plot depicts how the period of oscillations in the Selkov-PFB network fluctuates with temperature when the two reaction rates are temperature compensated (as specified in the legend) and all others are allowed to respond to temperature change. The analysis is done for all of the possible reaction rate combinations associated with this model. Initial concentrations of the molecules and the parameters are listed in Supplementary Table 1D.

Supplementary Table 1: Parameters

The activation energy for each reaction (in the main text, methods section), initial concentrations, and the rate constant values at 298 K, are given below in the Table 1. Table 1 has subdivided into 5 tables: Table 1A (for cyano-KaiABC network), Table 1B (for Two-Variable-Goodwin-NFB network), Table 1C (for cPNFB network),Table 1D (for Selkov-PFB network) and Table 1E (for Selkov-like PFB based model with an additional NFB loop).

Supplementary Table 1A. Parameters for cyano-KaiABC Network

**i.** Activation Energy (E) in KJ/mol				
$\mathbf{k_{half}}$	$\mathbf{k_{UT}^{A}}$	$\mathbf{k_{DT}^{A}}$	$\mathbf{k_{TU}^{0}}$	$\mathbf{k_{TU}^{A}}$
16.83	16.57	19.09	18.61	21.00
$\mathbf{k_{SD}^{A}}$	$\mathbf{k_{DS}^{0}}$	$\mathbf{k_{US}^{A}}$	$\mathbf{k_{SU}^{0}}$	$\mathbf{k_{TD}^{A}}$
16.43	17.64	22.01	20.21	18.57
Rates (A.U)				
$\mathbf{k_{UT}^{0}}$	$\mathbf{k_{DT}^{0}}$	$\mathbf{k_{TD}^{0}}$	$\mathbf{k_{SD}^{0}}$	$\mathbf{k_{US}^{0}}$
0	0	0	0	0

**ii.** Activation Energy (E) in KJ/mol	
$\mathbf{k_{DS}^{A}}$	$\mathbf{k_{SU}^{A}}$
17.57	19.74

Rust et al [1] proposed some negative rates in for their model. To handle these, the Arrhenius equation had to be updated as:

$rate = A_e \cdot (-e^{\frac{-E}{R \cdot T}})$ (shaded boxes above, Table 1A-ii)

**iii.** Initial Concentration (A.U)				
**KaiC**	**KaiA**	**T**	**ST**	**S**
3.4	1.3	0.68	1.36	0.34

The actual reaction rate for the cyano-KaiABC network at temperature 298 K can be represented as follows:

iv. Reaction Rate (A.U) at temperature 298 K								
k_{half}	k_{UT}^{A}	k_{DT}^{A}	k_{TU}^{0}	k_{TU}^{A}	k_{SD}^{A}	k_{DS}^{0}	k_{US}^{A}	k_{SU}^{0}
0.4302	0.4793	0.1731	0.2101	0.0799	0.5059	0.3101	0.0533	0.11
k_{TD}^{A}	k_{UT}^{0}	k_{DT}^{0}	k_{TD}^{0}	k_{SD}^{0}	k_{US}^{0}	k_{DS}^{A}	k_{SU}^{A}	
0.2130	0	0	0	0	0	-0.3195	-0.1331	

Supplementary Table 1B. Parameters for Two-Variable-Goodwin-NFB Network

i. Activation Energy (E) in KJ/mol						
k_1	k_2	γ_1	γ_2	k	α_1	α_2
31.8565	31.8565	14.7420	14.7420	12	14.01	17.9420

The actual reaction rate for the Two-Variable-Goodwin-NFB [2] network at temperature 298 K can be represented as follows:

ii. Reaction Rate (A.U) at temperature 298 K						
k_1	k_2	γ_1	γ_2	k	α_1	α_2
0.001	0.001	1.0003	1.0003	3.0253	1.3442	0.2749

iii. Initial Concentration (A.U)	
X	Y
1	0

Supplementary Table 1C. Parameters for cPNFB Network

i. Activation Energy (E) in KJ/mol							
p_0	p_3	d_1	d_2	k_{01}	k_{02}	k_{03}	k_{04}
6.45	10	10	6.45	21.55	21.55	21.55	21.55

The actual reaction rate for the cPNFB network [3] at temperature 298 K can be represented as follows:

ii. Reaction Rate (A.U) at temperature 298 K							
p_0	p_3	d_1	d_2	k_{01}	k_{02}	k_{03}	k_{04}
28.417	6.7816	6.7816	28.417	0.0641	0.0641	0.0641	0.0641

iii. Initial Concentrations (A.U)					
**OO**	**PP**	**OP**	**PO**	**B**	**A**
2	1	0	0	2	1

Supplementary Table 1D. Parameters for Selkov-PFB Network

i. Activation Energy (E) in KJ/mol			
**k_1**	**k_2**	**k_3**	**k_4**
21.5	27.5	5.1768	14.8691

The actual reaction rate for the Selkov-PFB network [4] at temperature 298 K can be represented as follows:

ii. Reaction Rate (A.U) at temperature 298 K			
**k_1**	**k_2**	**k_3**	**k_4**
0.0654	0.0058	47.5057	0.9503

iii. Initial Concentration (A.U)	
**Y**	**U**
0.48	0.0075

Supplementary Table 1E. Selkov-like PFB based model with an additional NFB loop

Cases	i. Activation Energy (E) in KJ/mol				
**Small k_3 - Large k_5**	**k_1**	**k_2**	**k_3**	**k_4**	**k_5**
	16.35	27.5	12.0203	14.8691	5.17
**Large k_3 - Small k_5**	**k_1**	**k_2**	**k_3**	**k_4**	**k_5**
	21.5	27.5	5.17	14.8691	12.0203
**Large k_3 - Large k_5**	**k_1**	**k_2**	**k_3**	**k_4**	**k_5**
	20.877	27.5	5.17	14.8691	5.17

Cases	ii. Reaction Rate (A.U) at temperature 298 K				
**Small k_3 - Large k_5**	**k_1**	**k_2**	**k_3**	**k_4**	**k_5**
	0.2325	0.0058	3	0.9503	47.63
**Large k_3 - Small k_5**	**k_1**	**k_2**	**k_3**	**k_4**	**k_5**
	0.0691	0.0058	47.63	0.9503	3

Large k_3 - Large k_5	k_1	k_2	k_3	k_4	k_5
	0.0841	0.0058	47.63	0.9503	47.63

**iii.** Initial Concentration (A.U)	
**Y**	**U**
0.48	0.0075

Supplementary Note 1. Addition of noise to the models

For equal handling of all models, we chose 1000 random pre-exponential factors (A_e) from a log normal distribution with a mean of 383.83 (A.U) and a variance of 30 ($(A.U)^2$) for all rates in each models. MATLAB code (https://www.mathworks.com/help/stats/lognrnd.html) for this purpose is shown below:

```
% Log-Normal Distribution with Variance = 30 (A.U)^2 and mean = 383.83 (A.U)
m = 383.83;  % mean value of Ae
v = 30;   % variance of Ae
mu = log((m^2)/sqrt(v+m^2))  % mean of the logarithmic values
sigma = sqrt(log(v/(m^2)+1))  % standard deviation of the logarithmic values
% For generating random numbers
r = lognrnd(mu,sigma,[1000, 1]);
% end
```

In the Supplementary Figure 9 we have displayed the multiplicative factors to the pre-exponential values that creates randomness to the reaction rates. For the purposes of calculating these multiplicative factors, we used the ratio of these randomly picked pre-exponential values to the mean pre-exponential value. Smaller variations in these multiplicative factors (mean ± standard deviation, 1 ± 0.0142) can build up to a big overall variation for all reaction rates.

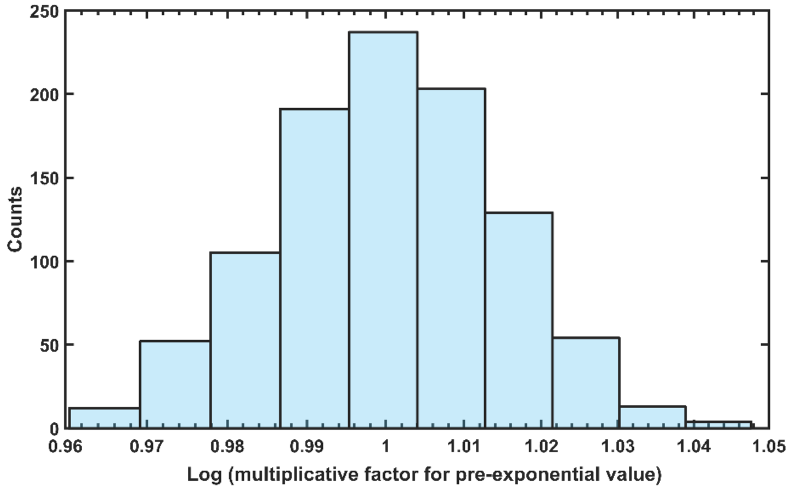

Supplementary Figure 9. A histogram of multiplicative factor for pre-exponential values chosen at random from a log normal distribution. Pre-exponential (A_e) values are randomly selected from a log normal distribution. The multiplicative factors to the mean pre-exponential value for creating random numbers are displayed in the histogram above (with blue color) and are represented in a logarithmic scale.

Supplementary Note 2. Statistical Measurements

We have quantified the noise in terms of Bayesian Information Criterion (BIC) and Total Parameter Variation.

Supplementary Note 2A. Total Parameter Variation

We calculated total parameter variation, caused by random selection of parameters by calculating the arithmetic mean of the distances of parameters from the nominal value (labeled as *true* below) [5]:

$$Arithmetic\ Mean = \frac{\sum_{i=1}^{k} \left| \dfrac{Rate_{random_i} - Rate_{true_i}}{Rate_{true_i}} \right|}{\mathrm{k}}$$

Where *k* = total number of parameters.

Because the arithmetic mean is stated as a ratio in this context, it is essentially a unitless quantity and its value depends on the number of parameters perturbed.

Supplementary Note 2B. Bayesian Information Criterion (BIC):

The BIC is a well-known generic model selection strategy that penalizes more complicated models by applying a cost based on the number of parameters being estimated in the model. A lower BIC value indicates a model that show lower deviations from the mean solution, when parameter

values are randomly chosen, compensating for the fact that larger models might be more noise resistant. We have calculated the BIC in the following manner:

$$BIC = n \cdot \log(SSE) - n \cdot \log(n) + k \cdot \log(n)$$

Where, n = sample size (we have considered *n* =200 random parameter sets in a limited total parameter variation regime), *k* = total number of parameter in a model and *SSE* = sum squared error. We have estimated the *SSE* as the following:

$$SSE = \sum_{i=1}^{n} (Y_{random} - Y_{average})^2$$

Supplementary Table 2. Total Number of Parameters

**Model**	**Total number of Parameters (*k*)**
cyano-KaiABC Network	12
Goodwin-NFB Model	7
cPNFB Network	8
Selkov-PFB Model	4
Selkov-like PFB based model with an additional NFB loop	5

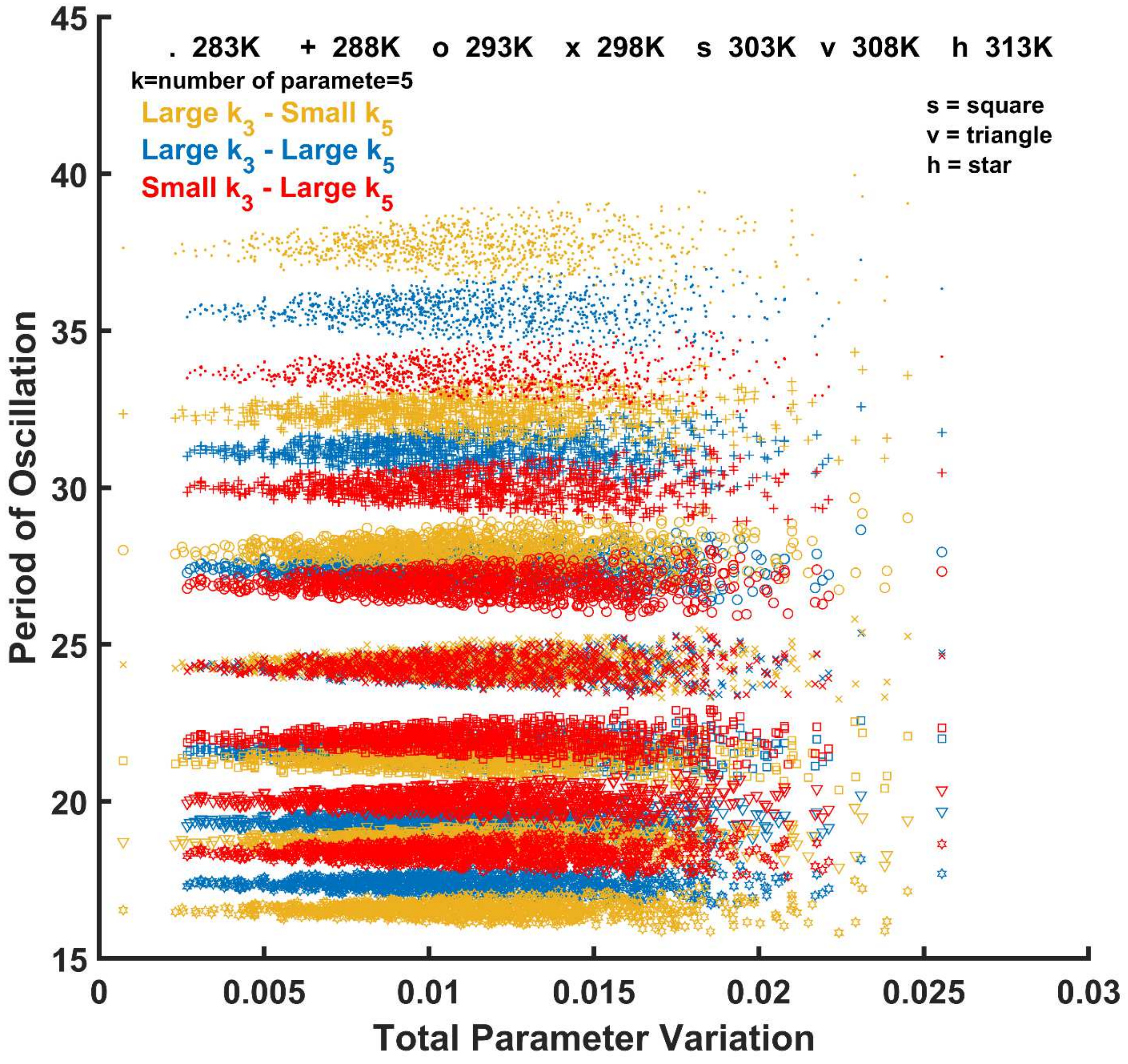

Supplementary Figure 10. The relationship between total parameter variations and oscillation period for a Selkov-like PFB based model with an additional NFB loop. The figure depicts how the period of oscillations varies in proportion to total parameter changes (Supplementary Note 2A) for one thousand random sample points at seven different temperatures in an oscillatory network (Figure 6a). The three different cases (Methods section, main text) corresponding to the network (Figure 6a) are distinguished by three separate colors, and the distinct seven temperatures are marked by different shaped markers. Initial concentrations of the molecules and the parameters are listed in the Supplementary Table 1E.

Supplementary References

1. Rust, M. J., Markson, J. S., Lane, W. S., Fisher, D. S. & O'Shea, E. K. Ordered phosphorylation governs oscillation of a three-protein circadian clock. *Science* **318**, 809–812 (2007).
2. Gonze, D. & Ruoff, P. The Goodwin Oscillator and its Legacy. *Acta Biotheor.* **69**, 857–874 (2021).
3. Hernansaiz-Ballesteros, R. D., Cardelli, L. & Csikász-Nagy, A. Single molecules can operate as primitive biological sensors, switches and oscillators. *BMC Syst. Biol.* **12**, 70 (2018).
4. SEL'KOV, E. E. Self-Oscillations in Glycolysis 1. A Simple Kinetic Model. *Eur. J. Biochem.* **4**, 79–86 (1968).
5. Barkai, N. & Leibler, S. Robustness in simple biochemical networks. *Nature* **387**, 913–917 (1997).
